# Research trends of inflammation in autism spectrum disorders: a bibliometric analysis

**DOI:** 10.3389/fimmu.2025.1534660

**Published:** 2025-02-14

**Authors:** Yajing Chen, Xiaohuan Du, Xianming Zhang, Fang Li, Shuwei Yuan, Wenjing Wang, Zengyan Zhu, Mei Wang, Chao Gu

**Affiliations:** Department of Pharmacy, Children’s Hospital of Soochow University, Suzhou, China

**Keywords:** autism spectrum disorder, inflammation, neuroinflammation, bibliometrics, citation analysis

## Abstract

**Background:**

Inflammation has been recognized as a significant factor in the pathophysiology of autism spectrum disorders (ASD), which have garnered increasing scholarly attention over the past few decades. This study aims to explore research trends related to inflammation and ASD through bibliometric analysis.

**Method:**

A comprehensive literature search was conducted in the Web of Science Core Collection (WoSCC) on August 28, 2024. This study was restricted to literature published in English. The bibliometric analysis utilized VOSviewer, CiteSpace, and the R package “bibliometrix” to visualize collaborations, keyword co-occurrences, and emerging research trends.

**Results:**

A total of 1,752 articles addressing inflammation and ASD were published, demonstrating a consistent upward trend in research output. The United States emerged as the country with the highest volume of publications. Saleh A. Bakheet was identified as the most prolific authors, significantly contributing to the literature with 54 publications. The University of California System was recognized as the most productive institution in this area of study. The journal of *Brain Behavior and Immunity* was noted as a prominent venue for publication in this field, exhibiting high citation metrics that reflect its considerable influence. The keyword “children” was the most frequently occurring term, with other significant terms including “oxidative stress” and “brain.” The keyword burst analysis revealed notable periods of increased research focus on topics such as “inflammatory bowel disease,” “cytokine production,” “neurodevelopmental disorders,” and “microbiota.”

**Conclusion:**

This bibliometric analysis highlights the growing scholarly attention devoted to the relationship between inflammation and ASD. Significant contributions and emerging trends emphasize the pivotal role of neuroinflammation in ASD, indicating a necessity for further exploration in this domain.

## Introduction

Autism spectrum disorders (ASD) encompass a spectrum of intricate neurodevelopmental conditions characterized by deficits in social communication, restricted interests, and repetitive behaviors (1). The global prevalence of ASD has risen in recent decades, with current estimates indicating a prevalence rate of 0.72% (2). Historically, treatment approaches for ASD have predominantly emphasized behavioral therapies (3), while pharmacological interventions are also utilized to address comorbid symptoms (4). Although these therapeutic modalities can mitigate certain symptoms, they do not adequately target the core deficits associated with ASD, and many children exhibit limited responsiveness to these interventions. The precise etiology of ASD remains elusive; however, research indicates a multifactorial origin that encompasses genetic, environmental, and neurobiological factors, including dysregulation of the immune system and inflammatory responses (5).

One key area of focus in ASD research is the role of inflammation in its pathophysiology. Neuroinflammation, particularly involving dysregulation of the immune system, has increasingly been linked to ASD, suggesting that immune responses may play a critical role in the onset and progression of the disorder (6). Bryn et al. reported significantly elevated levels of certain pro-inflammatory cytokines in children with ASD, alongside decreased levels of anti-inflammatory cytokines such as interleukin-10 (IL-10) (7). The activation of multiple inflammasome complexes in ASD, particularly interleukin-1 beta (IL-1β) and interleukin-18 (IL-18), is central to the inflammatory process. The activation of these inflammasomes leads to chronic inflammation, which is believed to exacerbate autistic behaviors (8). Given the growing interest in understanding the links between inflammation and ASD, it is essential to evaluate how this research field has evolved over time.

Bibliometrics is a quantitative analytical method employed to evaluate and measure the impact and trends of scientific publications, thereby offering insights into research dynamics, identifying emerging trends and influential contributions, and informing funding and policy decisions within the scientific community (9). Zhao et al. analyzed research trends in childhood ASD from 2012 to 2021, highlighting key topics such as coronavirus disease 2019, gut microbiota, and physical activity, with the United States leading in publication output (10). Rong et al. conducted a bibliometric study on ASD research spanning from 1998 to 2021, emphasizing trends in etiology, diagnostic markers, and the impact of COVID-19, while also identifying future directions, including case identification, large-scale cohort studies, and drug trials (11). Shen et al. conducted a bibliometric analysis of studies on neuroinflammation in ASD from 2004 to 2021, highlighting recent attention on short-chain fatty acids, mast cells, glial cells, and the future importance of gut microbiota and immune system research (12). Although research on neuroinflammation in ASD has been expanding, broader systemic inflammation—beyond neuroinflammation—remains underexplored despite its significant role in the pathophysiology of ASD. This gap highlights the necessity for a dedicated analysis of the inflammatory processes associated with ASD. To the best of our knowledge, no bibliometric analysis has specifically examined the relationship between systemic inflammation and ASD.

The aim of this study is to conduct a comprehensive bibliometric analysis of research trends concerning inflammation and ASD from 1994 to 2024, with a focus on literature published in English. By identifying key research themes, influential publications, and emerging areas of interest, this study will provide valuable insights into the role of inflammation in ASD, contributing to a more holistic understanding of the disorder and informing potential future therapeutic strategies.

## Materials and methods

### Search strategies and data collection

A literature search was conducted utilizing the Web of Science Core Collection (WoSCC) to investigate inflammation and ASD from 1994 to 2024. The WoSCC, recognized as a comprehensive and authoritative database, provides access to high-quality academic publications across various disciplines (13). The search formula employed was: (TS=(Inflammat*)) AND TS=(“Autism Spectrum Disorder” OR “autistic*” OR “autism*” OR “ASD” OR “Kanner*”) (10, 11, 14, 15). Only publications in the English language were included, with a concentration exclusively on articles among the various document types. Document types such as review articles, meeting abstracts, editorial materials, early access publications, letters to the editor, proceedings papers, book chapters, retractions, corrections, data papers, news items, publications with expressions of concern, and reprints were excluded. To minimize discrepancies arising from database updates, literature retrieval was executed on August 28, 2024. Bibliographic information was exported utilizing the “Full record and cited references” and “plain text” formats during the filtering process. Data were collected in text format, encompassing publication and citation counts, titles, author details, institutions, countries/regions, keywords, and journals for subsequent bibliometric analyses. The formulation of the search strategy was initially conducted independently by two reviewers, each developing their own search criteria. After reviewing the initial results, both reviewers consulted relevant high-quality literature to evaluate and refine their strategies. The final search methodology was determined through discussion and mutual agreement based on this evaluation. The screening process was carried out by two independent reviewers.

### Statistical analysis

Three robust bibliometric tools were utilized for the visualization and comprehensive analysis of academic data: VOSviewer (version 1.6.20), CiteSpace (version 6.3.R1), and R package “bibliometrix”. VOSviewer, recognized as versatile software, was pivotal in mapping institutional and author collaborations, co-authorship, citation patterns, keyword co-occurrence network, and co-citation networks (16). This tool enabled the visualization and exploration of intricate collaborative networks within the academic domain, providing profound insights into the interconnections among authors, institutions, and publications.

To enhance the comprehension of emerging trends and research hotspots, CiteSpace was utilized to identify keyword bursts. The parameters were established as follows: time slicing was defined from January 1994 to July 2024, with keywords designated as the selected node type. The threshold for keyword nodes was established at 5 prior to each fragment, with pruning performed utilizing the pathfinder and clip merge network methods. Visualization analysis was conducted according to these parameters to generate a timeline of keywords within the research field concerning inflammation and ASD.

The R package “bibliometrix” (version 3.2.1) was employed for a comprehensive bibliometric analysis. This tool was essential in analyzing and mapping the global distribution of research output, identifying significant trends and patterns, and evaluating the impact of authors, journals, and institutions within the dataset. The H-index was employed to quantify the academic impact of both individuals and journals, providing a balanced metric of academic influence (17, 18). Journal Citation Reports (JCR) quartiles and Impact Factor (IF) were utilized to evaluate the prestige and citation influence of journals. JCR quartiles categorize journals into four tiers, with Q1 representing the highest level of academic impact, while the IF quantifies the average number of citations received by articles published in a journal over the preceding two years. The most recent 2024 release of the JCR and IF data was utilized to ensure a contemporary assessment of journal prestige and citation influence.

## Results

### An overview of publications

**Figure 1:**
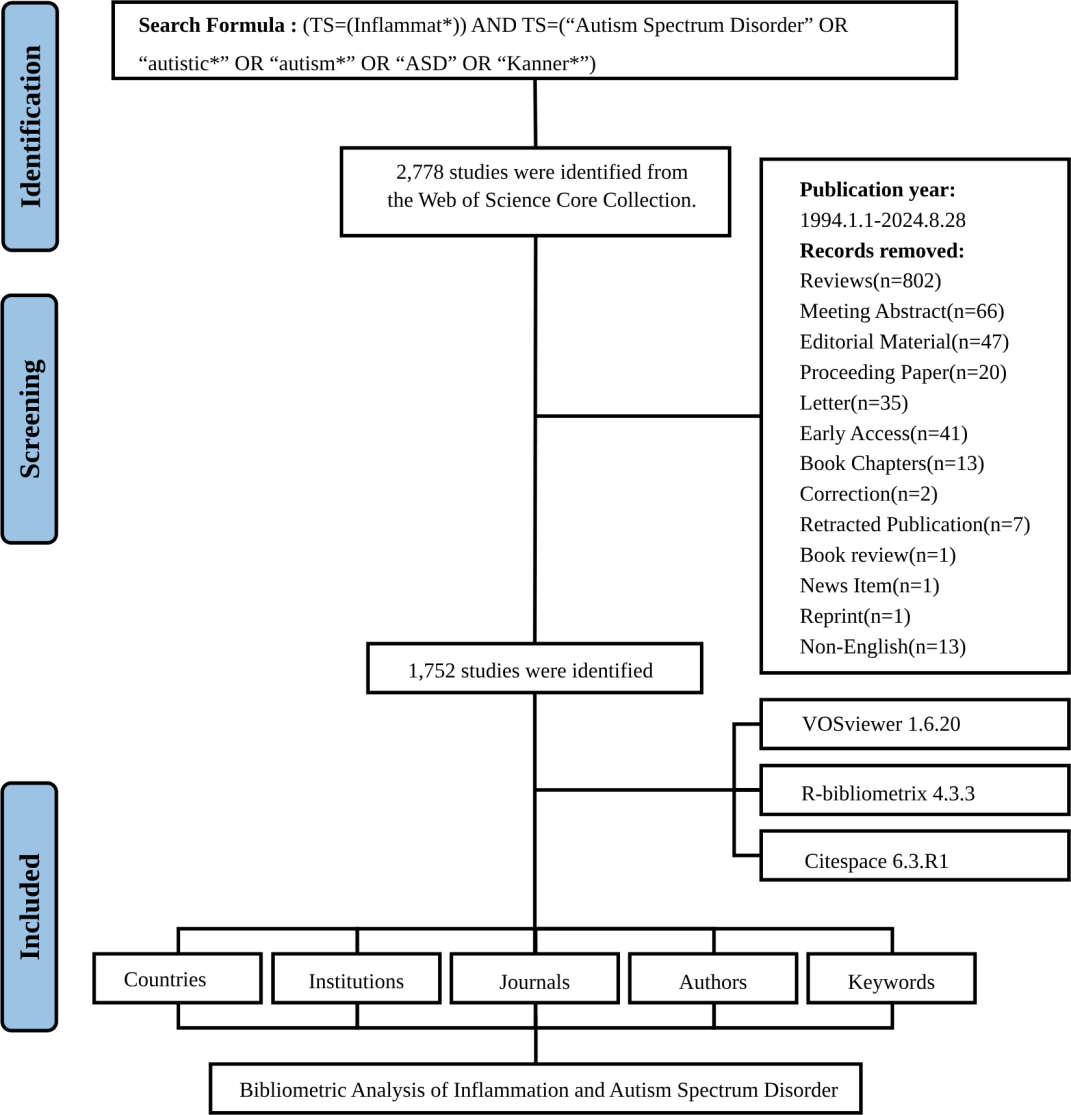
Flowchart of the literature screening process.

The flowchart depicting the data screening process was presented in **Figure 1**. The investigation revealed that 10,193 authors from 7,109 institutions across 507 countries contributed to the production of 1,752 manuscripts included in this study. These works were published in 635 journals, citing a total of 73,526 references.

**Figure 2:**
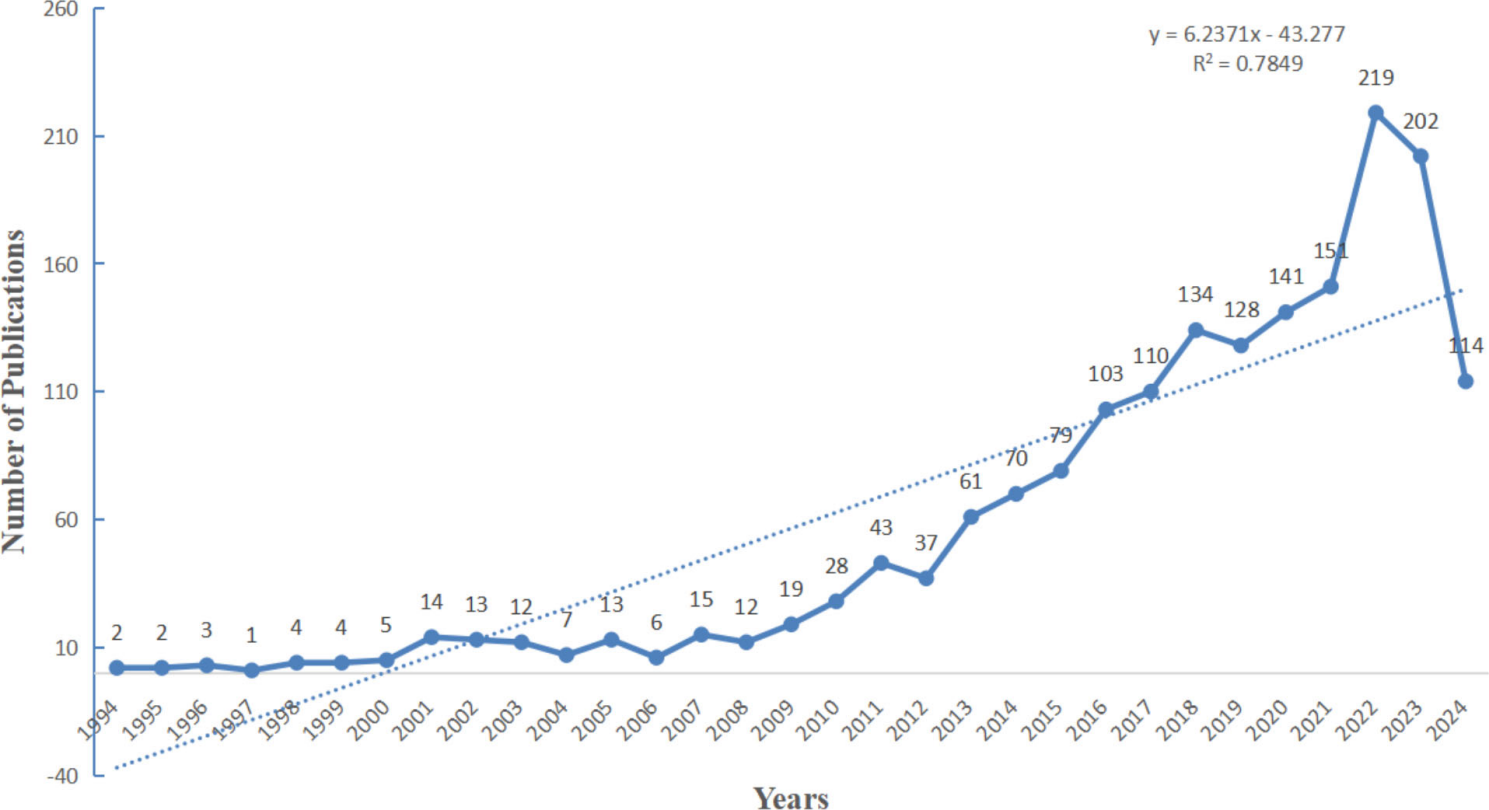
Overview of publications in research.

As depicted in **Figure 2**, the annual number of publications related to research on inflammation and ASD had exhibited a consistent upward trend over the years. Beginning with a modest count in the mid-1990s, the field began to gather momentum around 2010, with a gradual increase in publications each year. The period from 2012 to 2021 experienced a particularly pronounced rise, with the number of publications climbing steadily, reflecting heightened interest and significant advancements in this area of research. As of 2024, 114 articles have already been published, indicating that the upward trend in research on this topic is likely to continue and attract sustained attention in the future.

The most frequently cited study in this field was “Neuroglial Activation and Neuroinflammation in the Brain of Patients with Autism,” published in 2005 in the *Annals of Neurology* (IF: 8.1). This foundational work, which garnered 1,498 citations, underscored the role of neuroinflammation in ASD and had significantly influenced subsequent research (19). Another pivotal publication was “How Informative is the Mouse for Human Gut Microbiota Research?” published in 2015 in *Disease Models & Mechanisms* (IF: 4.0), which accumulated 853 citations (20). Furthermore, the third most cited study, “Gastrointestinal Flora and Gastrointestinal Status in Children with Autism—Comparisons to Typical Children and Correlation with Autism Severity,” published in 2011 in *BMC Gastroenterology* (IF: 2.5), received 660 citations (21).

### Analysis of the countries

**Figure 3:**
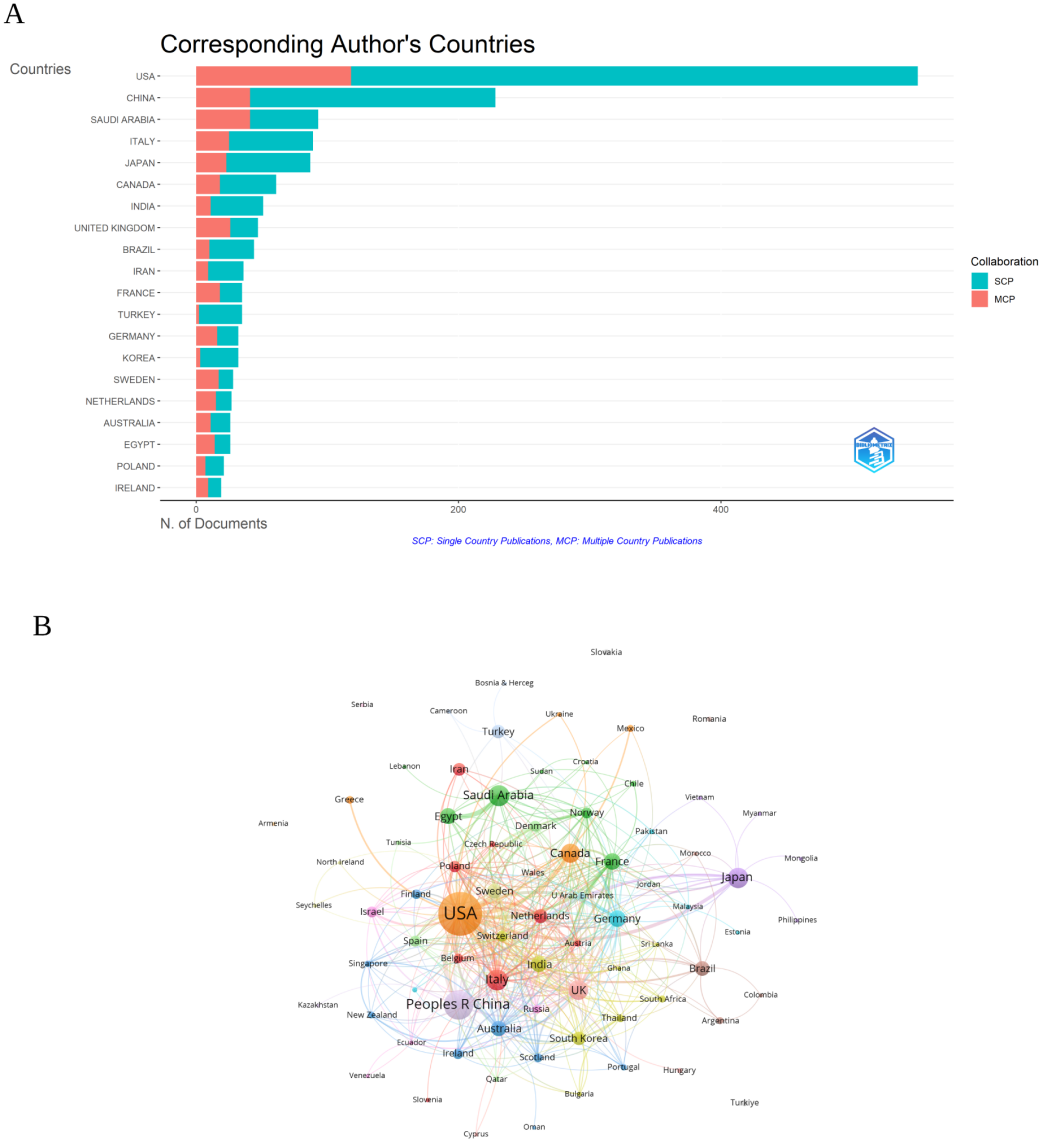
**(A)** Distribution of corresponding author’s publications by country. SCP, Single Country Publications; MCP, Multiple Country Publications. **(B)** Visualization map depicting the collaboration among different countries. (VOSViewer).

As illustrated in **Figure 3A** and detailed in **Table 1**, the leading countries in the field of research on inflammation and ASD have shown varying levels of productivity and international collaboration. The United States led significantly with 550 publications, representing the highest total publications (TP) (2,614) and topping the charts in total citations (TC) (29,565), with an impressive average of 53.8 citations per publication. China followed with 228 publications, ranking second in TP (977) and third in TC (3,570), with an average of 15.7 citations per article.

**Table 1:** Publication and citation profiles of leading countries.

Country	Articles	Freq	MCP_Ratio	TP	TP_rank	TC	TC_rank	Average Citations
USA	550	31.39269	21.45455	2614	1	29565	1	53.8
CHINA	228	13.0137	17.98246	977	2	3570	3	15.7
SAUDI ARABIA	93	5.308219	44.08602	321	5	2019	5	21.7
ITALY	89	5.079909	28.08989	460	3	3799	2	42.7
JAPAN	87	4.965753	26.43678	439	4	1874	7	21.5
CANADA	61	3.481735	29.5082	257	6	1950	6	32
INDIA	51	2.910959	21.56863	145	14	1088	13	21.3
UNITED KINGDOM	47	2.682648	55.31915	232	7	3079	4	65.5
BRAZIL	44	2.511416	22.72727	161	10	1075	14	24.4
IRAN	36	2.054795	25	151	12	966	15	26.8
FRANCE	35	1.997717	51.42857	203	8	415	23	11.9
TURKEY	35	1.997717	5.714286	145	15	335	26	9.6
GERMANY	32	1.826484	50	154	11	1526	10	47.7
KOREA	32	1.826484	9.375	123	17	620	19	19.4
SWEDEN	28	1.598174	60.71429	150	13	738	17	26.4
NETHERLANDS	27	1.541096	55.55556	133	16	824	16	30.5
AUSTRALIA	26	1.484018	42.30769	184	9	1631	8	62.7
EGYPT	26	1.484018	53.84615	117	18	329	27	12.7
POLAND	21	1.19863	33.33333	75	22	529	21	25.2
IRELAND	19	1.084475	47.36842	77	21	517	22	27.2

Articles, Publications of Corresponding Authors only; Freq, Frequence of Total Publications; MCP_Ratio, Proportion of Multiple Country Publications; TP, Total Publications; TP_rank, Rank of Total Publications; TC, Total Citations; TC_rank, Rank of Total Citations; Average Citations, The average number of citations per publication.

Saudi Arabia also stood out with 93 publications, ranking fifth in TP (321) but exhibiting a substantial multiple country publications (MCP) ratio of 44.09%, reflecting strong international collaboration. Italy and Japan were notable as well, with 89 and 87 publications respectively, both contributing significantly to the field with Italy achieving the second-highest TC (3,799) and Japan ranked seventh (1,874).

The ratio of MCP to single country publications (SCP) highlighted the varying levels of international collaboration among these countries. The United Kingdom and Sweden, for instance, had high MCP ratios of 55.32% and 60.71% respectively, indicating a strong emphasis on international research partnerships. In contrast, China, despite its large volume of publications, had a relatively lower MCP ratio of 17.98%, suggesting a more domestically-oriented research focus.

The visualization map illustrating collaboration among different countries in the research on inflammation and ASD was presented in **Figure 3B**. The United States was shown to have the highest number of collaborations, with 701 documents and a total citation count of 34,852, reflected in a strong total link strength of 413. The United Kingdom, with 104 documents and 4,990 citations, followed with a total link strength of 186, demonstrating significant collaborative efforts. China, represented by 263 documents and 4,545 citations, also displayed considerable international collaboration, with a total link strength of 126.

### Analysis of the institutions

**Figure 4:**
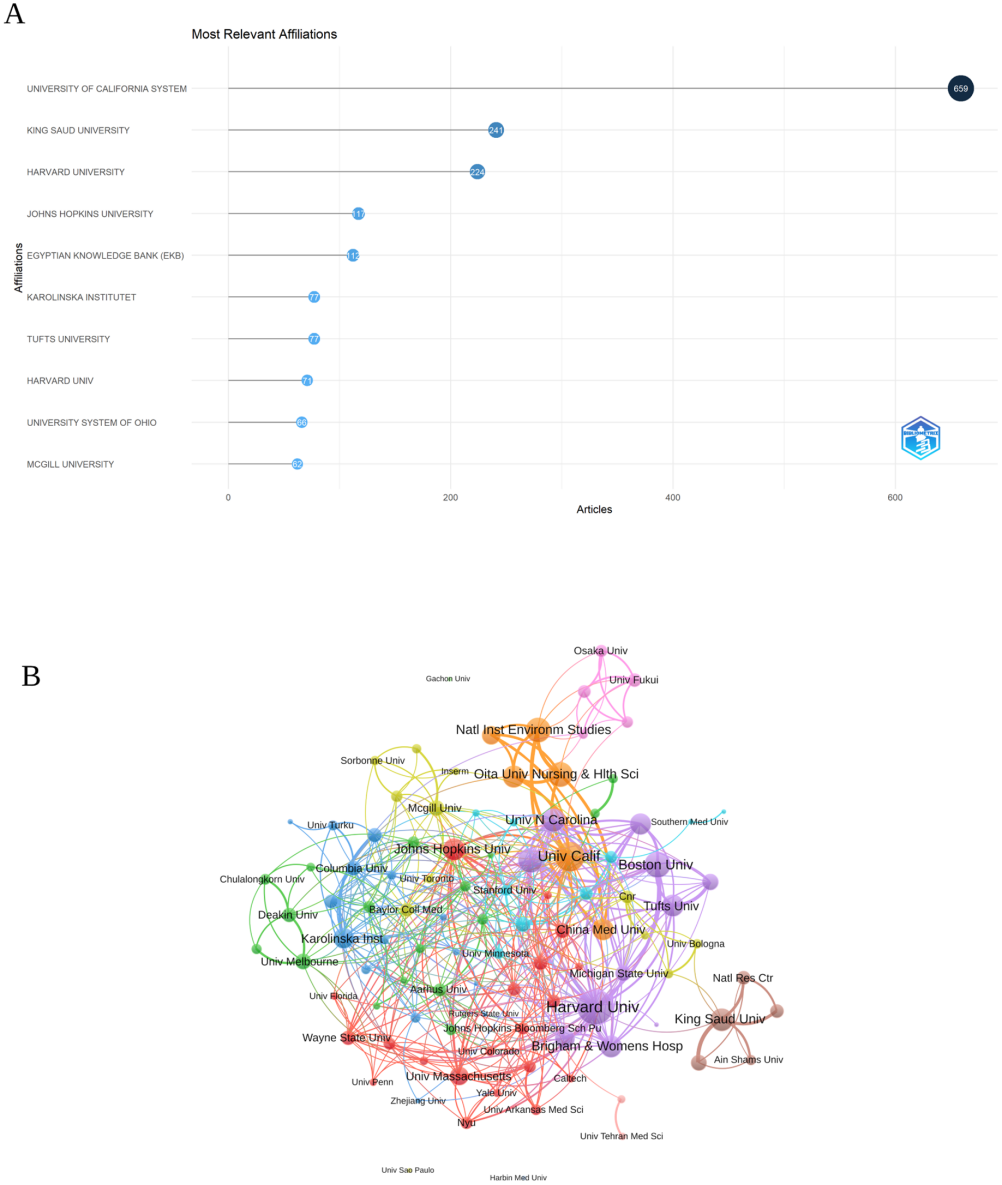
**(A)** Top ten institutions by article count and rank Circle size shows article count. Darker shades indicate higher ranks. **(B)** Visualization map depicting the collaboration among different institutions. (VOSViewer).

In **Figure 4A**, the top ten institutions are displayed according to their article count and rank in research related to inflammation and ASD. The University of California System was the leading institution, with 659 articles, reflecting its prominent role in advancing research in this area. King Saud University followed closely with 241 articles, indicating its significant contributions to the field. Harvard University also stood out, with 224 articles, demonstrating its strong involvement in the research community.

Among the 104 institutions engaged in international collaborations involving at least 8 articles, **Figure 4B** illustrates the collaboration among various institutions involved in research related to inflammation and ASD. Harvard University emerged as a prominent institution, with a total link strength of 136, reflecting its extensive collaborative network within this research area. The University of California also played a significant role, with a total link strength of 91, highlighting its strong partnerships and influence in the field. Boston University, though smaller in scale with 21 publications, had a notable total link strength of 73.

### Analysis of the authors

The top 20 high-impact authors in research related to inflammation and ASD are detailed in **Table 2**. Saleh A. Bakheet led with 54 publications, earning the highest total publication rank and a notable h-index of 23, accompanied by 396 total citations, ranking fourth in citation count. Ahmed Nadeem closely followed with 53 publications, an h-index of 23, and 395 total citations, placing him fifth in citation rank, indicating his significant contributions to the field. F. Ahmad Sheikh, also with 53 publications, achieved an h-index of 22 and ranks ninth in total citations with 380.

**Table 2:** Publication and citation profiles of high-impact authors.

Authors	h_index	g-index	m-index	PY_start	TP	TP_Frac	TP_rank	TC	TC_rank	Country
BAKHEET SALEH A.	23	34	2.555556	2016	54	6.753655	1	396	4	Saudi Arabia (2024)
NADEEM AHMED	23	34	2.555556	2016	53	6.586988	4	395	5	Saudi Arabia (2024)
AHMAD SHEIKH F.	22	33	2.75	2017	53	6.628655	2	380	9	Saudi Arabia (2024)
ASHWOOD PAUL	22	41	1.294118	2008	41	8.933896	5	693	1	USA (2024)
ATTIA SABRY M.	22	33	2.444444	2016	53	6.628655	3	389	7	Saudi Arabia (2024)
AL-AYADHI LAILA Y.	17	25	1.214286	2011	30	6.048846	7	183	19	Saudi Arabia (2024)
ANSARI MUSHTAQ A.	15	24	1.875	2017	34	4.175083	6	181	21	Saudi Arabia (2024)
VAN DE WATER JUDY	15	21	0.9375	2009	21	2.427255	10	485	2	USA (2024)
AL-HARBI NAIF O.	13	19	1.625	2017	19	2.453571	11	199	18	Saudi Arabia (2024)
EL-ANSARY AFAF	13	22	1	2012	29	6.521068	8	82	58	Saudi Arabia (2024)
HERTZ-PICCIOTTO IRVA	13	14	0.764706	2008	14	2.014683	17	463	3	USA (2024)
THEOHARIDES THEOHARIS C.	13	13	0.866667	2010	13	3.577381	20	86	53	USA (2024)
AL-AYADHI LAILA	11	18	0.846154	2012	18	4.827381	12	76	80	Saudi Arabia (2024)
ICHINOSE TAKAMICHI	11	20	0.6875	2009	22	3.245285	9	73	84	Japan (2024)
TAKANO HIROHISA	11	18	0.6875	2009	18	2.175444	13	66	119	Japan (2024)
YOSHIDA SEIICHI	11	13	0.6875	2009	13	1.378619	21	63	124	Japan (2019)
HE MIAO	10	13	0.769231	2012	13	1.535029	19	58	135	China (2019)
NISHIKAWA MASATAKA	10	16	0.625	2009	16	2.251696	14	67	109	Japan (2022)
SUN GUIFAN	10	12	0.769231	2012	12	1.368362	22	58	137	China (2019)
FRYE RICHARD E.	9	13	0.6	2010	13	2.973189	18	16	540	USA (2023)

H_index, The h-index of the journal, which measures both the productivity and citation impact of the publications; g_index, The g-index of the journal, which gives more weight to highly-cited articles; m_index, The m-index of the journal, which is the h-index divided by the number of years since the first published paper; TP, Total Publications; TP_rank, Rank of Total Publications; TC, Total Citations; TC_rank, Rank of Total Citations; Average Citations, The average number of citations per publication; PY_start, Publication Year Start, indicating the year the journal started publication; Country, Refers to the author’s most recent affiliation based on their latest publication in this research area.

**Figure 5:**
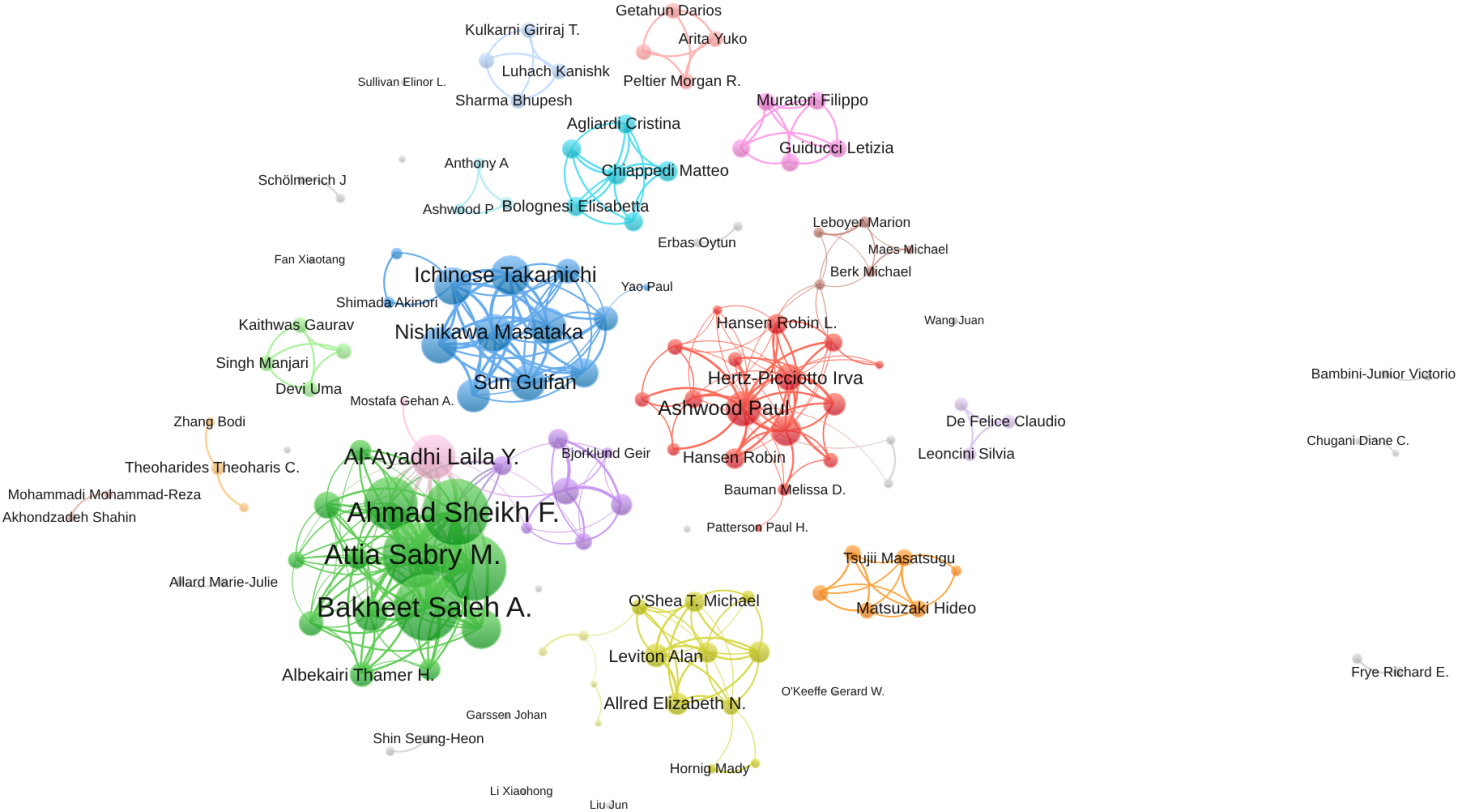
Visualization map depicting the collaboration among different authors. (VOSViewer).

In **Figure 5**, the collaboration network among 139 authors who are involved in international collaborations with a minimum of 5 articles is depicted, focusing on research related to inflammation and ASD. Bakheet Saleh A. had the highest number of collaborations with other countries with the total link strength of 287, indicating a robust and extensive network of international collaborations. Ahmad Sheikh F. followed closely with a total link strength of 284, reflecting significant collaborative efforts within the research community. Nadeem Ahmed and Attia Sabry M. also showed substantial collaboration, with total link strengths of 283 and 282, respectively. Among the 139 authors involved in international collaborations with a minimum of 5 articles, Bakheet Saleh A. had the highest number of collaborations with other countries (287), followed by Ahmad Sheikh F. (284) and Nadeem Ahmed (283).

### Analysis of journals

A comprehensive overview of the top 20 most productive journals in the field of inflammation and ASD was shown in **Table 3**. *Brain Behavior and Immunity* stood out as the leading journal, with an H-index of 42, an impact factor of 8.8, and a total of 92 publications, ranking first in both TP and TC (2,686), with an average of 29.2 citations per publication. *PLoS One* held the second position in TP with 37 publications and ranked second in TC (2,030), giving it the highest average of 54.9 citations per publication, which emphasized its significant impact on research despite having fewer total publications. *Journal of Neuroinflammation* ranks fourth in TP with 33 publications but was notable for its strong citation impact, ranking 12th in TC (1,014), with an average of 30.7 citations per publication.

**Table 3:** Top 20 most productive journals in the field of inflammation and autism spectrum disorders.

Journal	H_index	IF	JCR_Quartile	PY_start	TP	TP_rank	TC	TC_rank
BRAIN BEHAVIOR AND IMMUNITY	42	8.8	Q1	2010	92	1	2686	1
PLOS ONE	24	2.9	Q1	2008	37	2	2030	2
JOURNAL OF NEUROINFLAMMATION	23	9.3	Q1	2007	33	4	1014	12
TRANSLATIONAL PSYCHIATRY	18	5.8	Q1	2011	29	8	826	15
AUTISM RESEARCH	16	5.3	Q1	2013	28	9	685	20
MEDICAL HYPOTHESES	16	2.1	Q3	2001	30	7	223	87
BIOLOGICAL PSYCHIATRY	14	9.6	Q1	2007	14	18	1498	5
MOLECULAR PSYCHIATRY	14	9.6	Q1	2002	20	11	1440	6
PROGRESS IN NEURO-PSYCHOPHARMACOLOGY & BIOLOGICAL PSYCHIATRY	14	5.3	Q1	2015	17	15	519	31
SCIENTIFIC REPORTS	13	3.8	Q1	2016	33	5	761	17
FRONTIERS IN PSYCHIATRY	12	3.2	Q2	2015	30	6	387	45
INTERNATIONAL JOURNAL OF DEVELOPMENTAL NEUROSCIENCE	12	1.7	Q4	2011	18	12	377	46
JOURNAL OF NEUROSCIENCE	12	4.4	Q1	2006	14	19	1732	3
MOLECULAR AUTISM	12	6.3	Q1	2011	20	10	673	21
INTERNATIONAL JOURNAL OF MOLECULAR SCIENCES	10	4.9	Q2	2015	36	3	622	25
JOURNAL OF AUTISM AND DEVELOPMENTAL DISORDERS	10	3.2	Q1	2002	16	16	1577	4
JOURNAL OF NEUROIMMUNOLOGY	10	2.9	Q2	2006	18	13	1202	9
PEDIATRICS	10	6.2	Q1	2001	11	24	1079	10
BEHAVIOURAL BRAIN RESEARCH	9	2.6	Q3	2007	15	17	924	13
BRAIN SCIENCES	9	2.7	Q3	2019	13	20	115	181

H_index, The h-index of the journal, which measures both the productivity and citation impact of the publications; IF, Impact Factor, indicating the average number of citations to recent articles published in the journal; JCR_Quartile, The quartile ranking of the journal in the Journal Citation Reports, indicating the journal’s ranking relative to others in the same field (Q1, top 25%, Q2, 25%-50%, Q3, 50%-75%, Q4, bottom 25%); TP, Total Publications; TP_rank, Rank of Total Publications; TC, Total Citations; TC_rank, Rank of Total Citations; Average Citations, The average number of citations per publication; PY_start, Publication Year Start, indicating the year the journal started publication.

**Figure 6:**
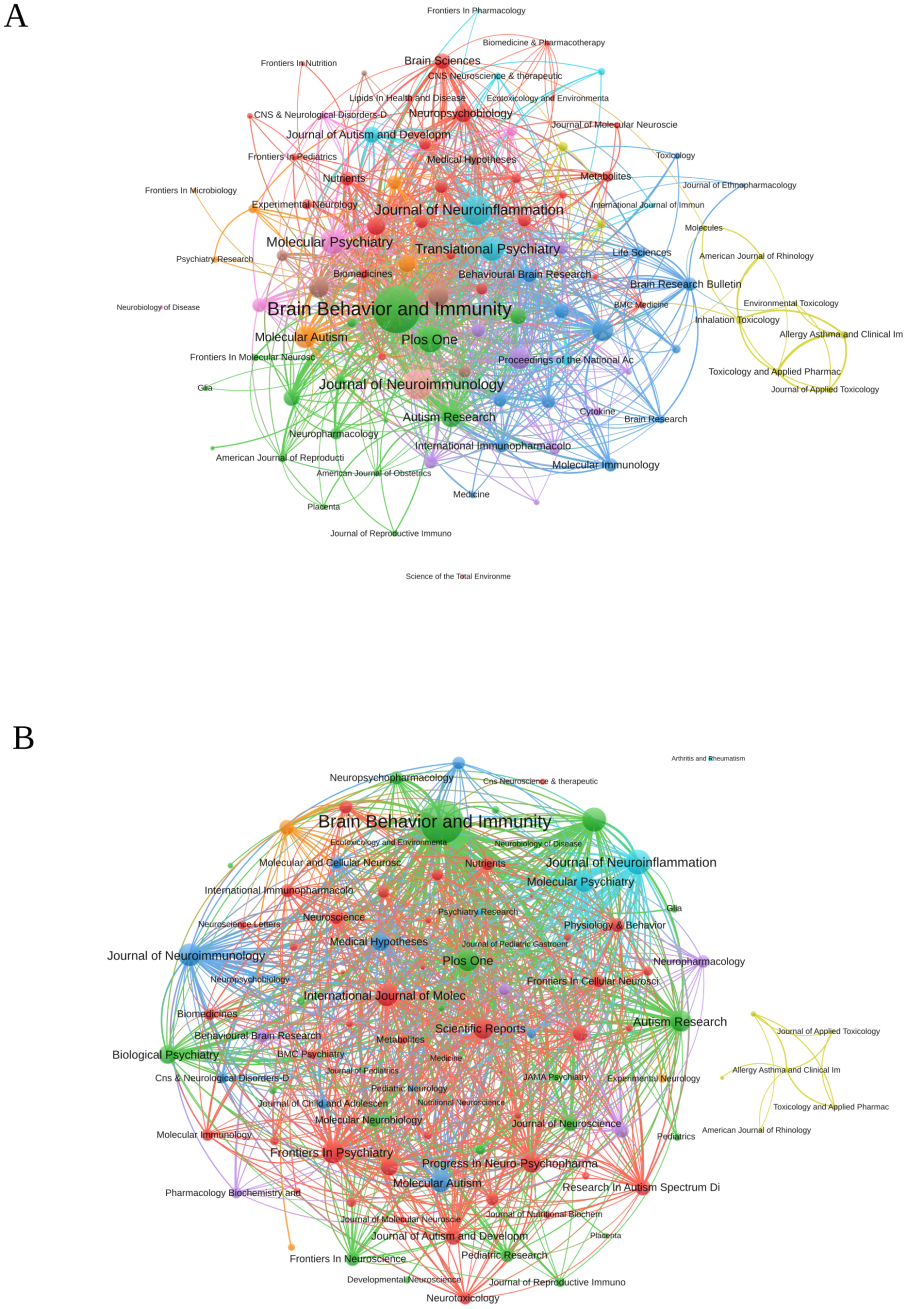
**(A)** Journal co-occurrence network diagram. **(B)** Journal bibliography coupling network diagram. (VOSViewer).

The co-occurrence network of journals related to research on inflammation and ASD is depicted in **Figure 6A**. The three key journals with the highest total link strength in these co-occurrence networks were *Brain Behavior and Immunity* with a total link strength of 889, *Journal of Neuroimmunology* with a total link strength of 362, and *Journal of Neuroinflammation* with a total link strength of 350. **Figure 6B** presented the journal coupling network diagram related to research. The three key journals with the highest total link strength in this coupling network were *Brain Behavior and Immunity* with a total link strength of 49,255, *Journal of Neuroinflammation* with a total link strength of 15,455, and *Translational Psychiatry* with a total link strength of 14,533.

### Analysis of the keywords

**Supplementary Table 1** highlighted the top 20 keywords in the field of inflammation and ASD. In this analysis, the keyword “children” emerged as the most frequently occurring term, with 382 occurrences and a total link strength of 1,302. Other prominent keywords included “brain” (308 occurrences, total link strength of 1,227), and “activation” (207 occurrences, total link strength of 805). Additionally, keywords such as “oxidative stress” (152 occurrences, total link strength of 552) further emphasized the broad range of factors explored in this area of research.

**Figure 7:**
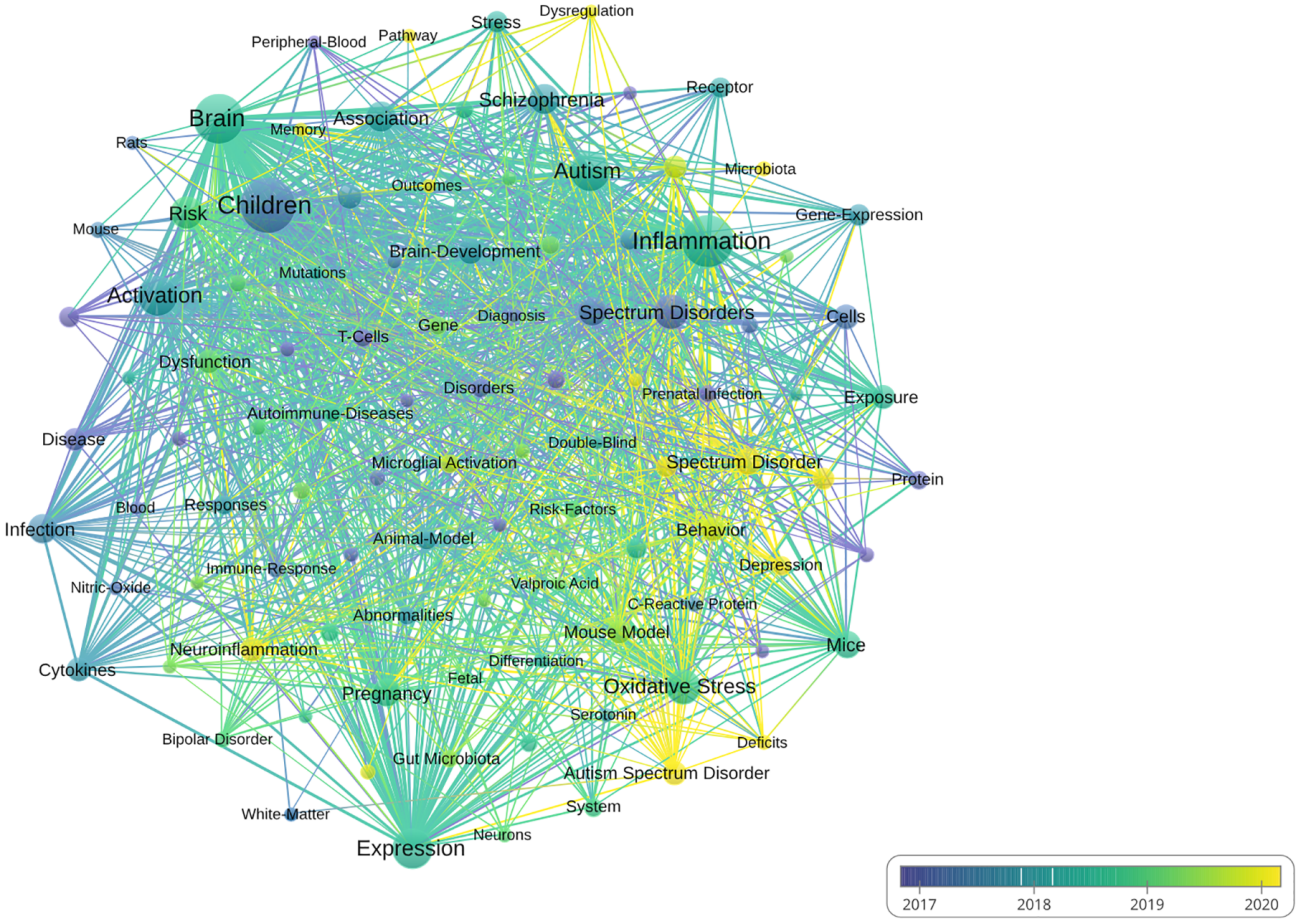
Visual analysis of keyword co-occurrence network analysis. (VOSViewer).

**Figure 7** presented a visual analysis of the keyword co-occurrence network in research related to inflammation and ASD. The first cluster focused on neurological aspects and included terms such as “brain” and “neuroinflammation,” both of which were key focal points in this area of study. The second cluster centered around immune and inflammatory responses, with related terms such as “activation” and “cytokine” highlighting the emphasis on understanding immune system dysregulation and its impact on ASD. The third cluster explored environmental and genetic influences, featuring keywords like “oxidative stress,” “prenatal infection,” and “spectrum disorders.”

### Analysis of burst keywords

**Figure 8:**
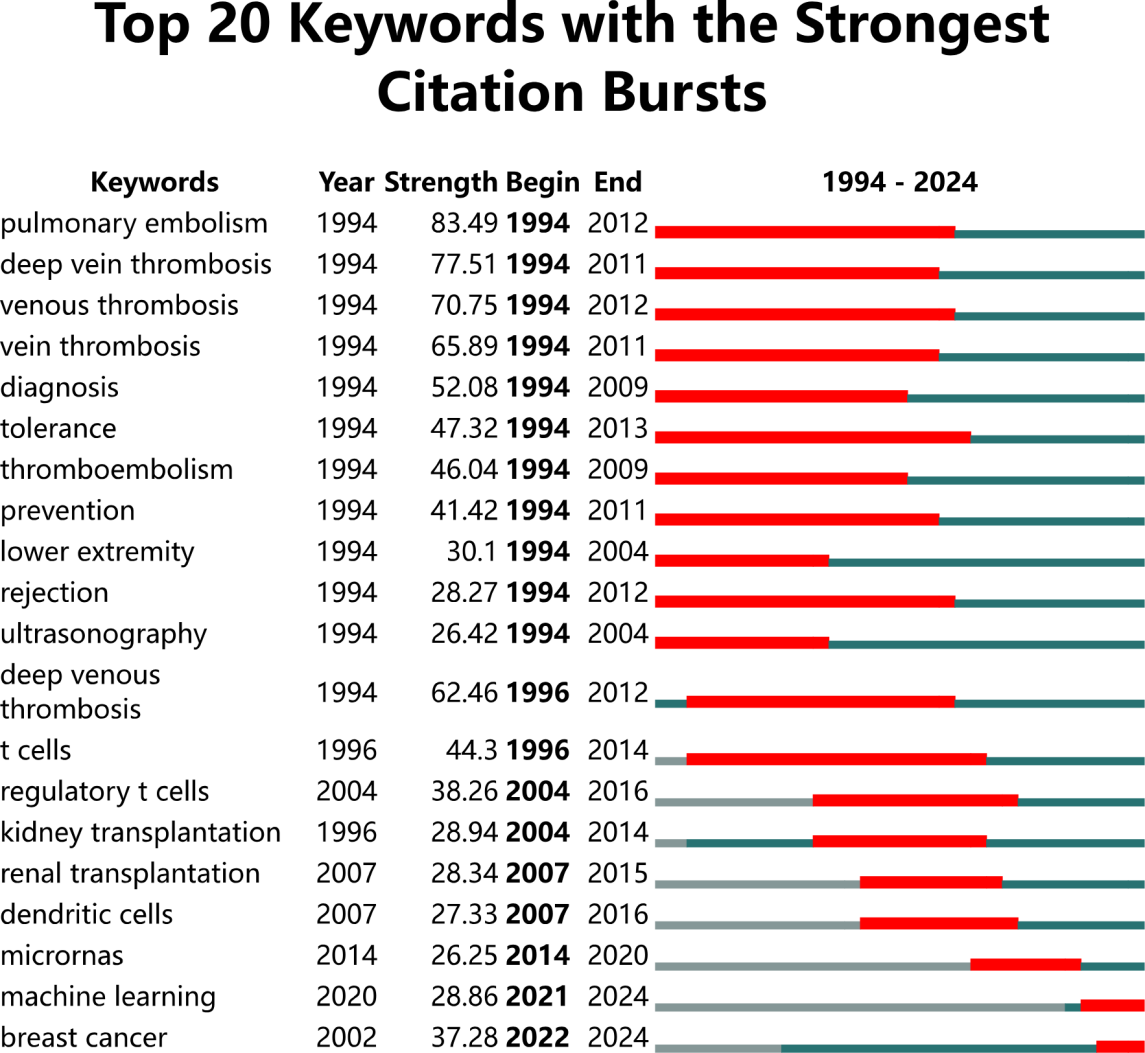
Top 20 Keywords with the strongest citation bursts.

A burst analysis of keywords revealed the evolving trends in research on inflammation and ASD (**Figure 8**). “Inflammatory bowel disease” experienced a strong citation burst from 2000 to 2011, indicating sustained interest in understanding its connection with systemic inflammation and potential links to neurodevelopmental disorders like autism. Similarly, “children” showed a notable burst from 2001 to 2013, highlighting the critical focus on pediatric studies related to ASD and inflammation. The keywords “cytokine production” (2005-2011) and “activation” (2009-2012) reflected a growing interest during this period in understanding the role of immune system dysregulation in ASD pathophysiology. More recent bursts, such as “NF Kappa B” (2017-2019) and “risk factors” (2019-2021), underscored a shift towards the molecular and genetic underpinnings of ASD. Keywords like “depression” (2020-2021) and “nitric oxide” (2020-2021) also reflected a recent focus on understanding comorbid conditions, particularly the overlap between ASD and other mental health issues. The recent burst of “neurodevelopmental disorders,” which started in 2021, suggested that current research was heavily focused on the intersection between immune responses and developmental brain disorders. The ongoing burst for “microbiota” (2022-2024) pointed to the increasing exploration of the gut-brain axis and its implications for ASD.

## Discussion

### General information

The bibliometric analysis highlights the expanding body of research exploring the intersection of inflammation and ASD, with a particular focus on neuroinflammation and immune dysregulation. A notable trend revealed by keyword burst analysis is the growing emphasis on “neurodevelopmental disorders” and “microbiota,” signaling a shift toward investigating the broader implications of immune responses on developmental outcomes in ASD. Shen et al. previously conducted a comprehensive bibliometric analysis of 620 articles in the field of neuroinflammation in autism, identifying research hotspots such as short-chain fatty acids, mast cells, and glial cells, and emphasizing the potential future research directions of gut microbiota and the immune system (12). Building on this foundation, our study extends the bibliometric scope to encompass the broader field of inflammation and ASD, including 1,752 relevant publications. Of particular note, 535 of these articles were published between 2022 and 2024, demonstrating a continuous upward trend in research output. The USA remains the dominant contributor to the literature, with Saleh A. Bakheet identified as the most prolific author and the University of California system as the leading institution. Our findings further underscore “oxidative stress” as a major research hotspot, with future research trends pointing toward “inflammatory bowel disease,” “cytokine production,” “neurodevelopmental disorders,” and “microbiota,” reflecting the substantial contribution of this study to advancing the understanding of inflammation in ASD.

The United States, particularly through institutions such as the University of California System, has played a dominant role, contributing to the highest number of publications and demonstrating significant academic influence through high citation metrics. This reflects the United States’ robust infrastructure for biomedical research and its ongoing investment in understanding neuroinflammatory processes in AUD (22). The United States has a strong culture of collaboration between academic institutions, hospitals, and research centers, facilitating interdisciplinary research. This allows for cutting-edge technological integration, such as neuroimaging, genomics, and bioinformatics, which are essential for studying inflammation in neurological contexts (23). The University of California System, with its multiple campuses and extensive research networks, exemplifies this integration, providing both the human resources and advanced facilities needed to drive high-impact research (24).

In terms of authorship, Saleh A. Bakheet emerged as one of the most prolific contributors to the field, with 54 publications and a high citation count, indicating his significant influence on the research landscape of inflammation and ASD. His work likely spans various key areas, including immune system dysregulation and neuroinflammation, both of which are central to understanding the complex interplay between ASD and inflammation. One of his studies found that elevated TLR4 expression in B cells of children with ASD is associated with increased oxidative stress, suggesting a potential contribution to systemic immune dysfunction in these individuals (25). This finding is of significant importance to the field as it highlights a specific immune mechanism that could be targeted for therapeutic intervention, advancing our understanding of how oxidative stress and immune dysregulation contribute to the pathophysiology of ASD.

### Emerging topics

The keyword co-occurrence network in research related to inflammation and ASD can be divided into three main clusters, reflecting different research themes: neurological aspects, immune and inflammatory responses, and environmental and genetic influences. The first cluster focuses on neurological aspects, with key terms such as “brain” and “neuroinflammation”. These keywords represent a significant research interest in understanding the neurological basis of ASD, particularly the role of neuroinflammation in brain development and function. Usui et al. suggests the roles of neuroinflammation and oxidative stress in the pathogenesis of ASD, emphasizing the impact of maternal immune activation as a significant environmental risk factor during pregnancy. Additionally, the review examines the potential benefits of anti-inflammatory drugs and antioxidants in both animal models and clinical studies, suggesting new avenues for therapeutic intervention (26). Matta et al. explores the significant role of dysregulated neuroinflammation in ASD, particularly the involvement of reactive microglia and astrocytes in immune responses and synaptic function. It notes the presence of increased reactive glial cells in ASD postmortem tissue and animal models, raising questions about how these changes may exacerbate alterations in neural connectivity (27). The second cluster is centered on immune and inflammatory responses, and associated terms such as “activation” and “cytokine” highlight the focus on immune system dysregulation and its contribution to ASD. This cluster underscores the growing body of research exploring how inflammatory processes, such as elevated levels of pro-inflammatory cytokines and immune activation, may exacerbate the severity of autism symptoms. Zhao et al. conducted a meta-analysis revealing that abnormal cytokine production, including increased levels of proinflammatory cytokines such as IL-6, is associated with ASD (28). Theoharides et al. suggested that cytokine production, particularly IL-1β and IL-6, increases blood-brain barrier permeability, which exacerbates neuroinflammation in ASD. These findings highlight the role of proinflammatory cytokines in contributing to neurological abnormalities in ASD (29). The third cluster encompasses environmental and genetic influences, with keywords like “oxidative stress,” and “prenatal infection,” representing this theme. These terms highlight research into how environmental factors, such as prenatal exposure to infections or toxins, interact with genetic predispositions to affect neurodevelopment in ASD. Chen et al. revealed significant oxidative stress marker aberrations in children with ASD. This study highlights that oxidative stress contributes to neurodevelopmental disorders, with inflammation being a potential mediator of ASD symptoms (30). The analysis of keyword bursts provides valuable insights into the evolving trends in research on inflammation and ASD.

### Research frontiers

In more recent years, starting from 2017, keywords such as “NF Kappa B” (2017-2019) and “risk factors” (2019-2021) highlighted a growing focus on the molecular and genetic underpinnings of ASD. NF-κB was found to be overactivated in children with ASD, which resulted in increased production of proinflammatory cytokines such as TNF-α. This upregulation of inflammatory mediators suggests that NF-κB contributes to the neuroimmune mechanisms of ASD (31). Environmental stressors may trigger NF-κB activation, which in turn drives the expression of inflammatory genes linked to ASD. This study suggests targeting NF-κB activation as a key therapeutic approach to managing inflammation-related ASD symptoms (32).

The bursts of “depression” and “nitric oxide” starting in 2020 represent emerging areas of comorbidity research, particularly in understanding the overlap between ASD and other mental health conditions such as depression. Robinson-Agramonte et al. explored the role of immune dysregulation in ASD, finding that elevated levels of pro-inflammatory cytokines not only contribute to ASD pathology but also predispose individuals to comorbid conditions such as depression and anxiety (33). Masi et al. discuss the role of neuroinflammation as a common mechanistic pathway between depression and ASD. They propose that anti-inflammatory treatments targeting cytokine production could alleviate both ASD symptoms and depression (34).

The recent burst in keywords like “microbiota” (2022-2024) reflects growing interest in the gut-brain axis and its potential influence on ASD pathology. Future research is likely to explore the role of gut microbiota in modulating immune responses and neurodevelopment in ASD. Emerging studies have shown that alterations in the composition of gut bacteria can lead to changes in neuroimmune signaling, which may exacerbate ASD symptoms. Hsiao et al. demonstrated that restoring healthy gut microbiota in mouse models of ASD led to significant improvements in behavior and immune function, suggesting a promising therapeutic avenue for managing ASD symptoms through microbiome interventions (35). As this area of research evolves, it is expected that future studies will focus on understanding the specific microbial strains and metabolites that contribute to the modulation of neuroimmune pathways, offering new opportunities for personalized treatment strategies.

### Strengths and limitations

This study has several strengths. First, it provides a comprehensive overview of research trends over three decades, offering valuable insights into the evolution of the field of inflammation and ASD. Second, the use of multiple robust bibliometric tools, including VOSviewer, CiteSpace, and “bibliometrix,” allowed for a detailed analysis of collaborations, keyword co-occurrences, and emerging research trends, enhancing the depth and accuracy of the findings. This study still has several limitations. First, the analysis was limited to articles published in English, which may have excluded relevant research published in other languages, potentially introducing language bias. Second, the citation analysis may have been influenced by the time lag in citation accumulation, potentially underrepresenting the impact of more recent publications.

## Conclusion

This bibliometric analysis systematically assesses the scope and evolution of research in the area of inflammation and ASD. The analysis highlights research hotspots centered on themes such as cytokine production. Emerging frontiers in the field indicate a growing emphasis on the complex relationship between neuroinflammation and ASD. The findings contribute to a deeper understanding of this evolving field and may have significant implications for clinical approaches to ASD.

## Data Availability

The original contributions presented in the study are included in the article/**Supplementary Material**. Further inquiries can be directed to the corresponding authors.
