# Supplementary material for: Research trends of inflammation in autism spectrum disorders: a bibliometric analysis
Source: Front Immunol. 2025 Feb 14;16:1534660. doi: 10.3389/fimmu.2025.1534660 (PMC11868081; doi:10.3389/fimmu.2025.1534660)
Supplement: Supplementary file 1 [file Table1.docx]

**Supplemental Table 1. Co-occurence analysis of Top 20 keywords.**

| Keyword | Occurrences | Total link strength |
| --- | --- | --- |
| children | 382 | 1302 |
| inflammation | 338 | 1249 |
| brain | 308 | 1227 |
| activation | 207 | 805 |
| autism | 201 | 776 |
| expression | 205 | 761 |
| oxidative stress | 152 | 552 |
| spectrum disorders | 147 | 520 |
| risk | 133 | 504 |
| schizophrenia | 116 | 491 |
| infection | 106 | 456 |
| pregnancy | 96 | 438 |
| association | 114 | 395 |
| mice | 92 | 393 |
| spectrum disorder | 93 | 355 |
| autism spectrum disorders | 108 | 338 |
| brain-development | 81 | 337 |
| cytokines | 71 | 325 |
| dysfunction | 69 | 312 |
| neuroinflammation | 71 | 307 |
